# Early signs of right ventricular systolic and diastolic dysfunction in acute severe respiratory failure: the importance of diastolic restrictive pattern

**DOI:** 10.1177/2048872619883399

**Published:** 2019-11-25

**Authors:** Guido Tavazzi, Niels Bergsland, Joana Alcada, Susanna Price

**Affiliations:** 1 Department of Clinical-Surgical, Diagnostic and Paediatric Sciences, University of Pavia, Italy; 2 Anesthesia and Intensive Care, Fondazione IRCCS Policlinico San Matteo, Italy; 3 Buffalo Neuroimaging Analysis Center, State University of New York, USA; 4 Adult Intensive Care Unit, Royal Brompton Hospital, London, UK; 5 Inflammation, Repair and Development, Imperial College London, UK

**Keywords:** Acute severe respiratory failure, right ventricular systo-diastolic function, echocardiography, right ventricular restrictive physiology

## Abstract

**Background:**

The incidence and pathophysiology of right ventricular failure in patients with severe respiratory insufficiency has been largely investigated. However, there is a lack of early signs suggesting right ventricular systolic and diastolic dysfunction prior to acute cor pulmonale development.

**Methods:**

We conducted a retrospective analytical cohort study of patients for acute respiratory distress syndrome undertaking an echocardiography during admission in the cardiothoracic intensive care unit. Patients were divided according to treatment: conventional protective ventilation (38 patients, 38%); interventional lung assist (23 patients, 23%); veno-venous extracorporeal membrane oxygenation (37 patients, 37%). Systolic and diastolic function was studied assessing, respectively: right ventricular systolic longitudinal function (tricuspid annular plane systolic excursion) and systolic contraction duration (tricuspid annular plane systolic excursion length); right ventricular diastolic filling time and right ventricular diastolic restrictive pattern (presence of pulmonary valve presystolic ejection wave). Correlation between the respiratory mechanics and systo-diastolic parameters were analysed.

**Results:**

In 98 patients studied, systolic dysfunction (tricuspid annular plane systolic excursion <16 mm) was present in 33.6% while diastolic restrictive pattern was present in 64%. A negative correlation was found between tricuspid annular plane systolic excursion and tricuspid annular plane systolic excursion length (*P*<0.0001; *r* −0.42). Tricuspid annular plane systolic excursion and tricuspid annular plane systolic excursion length correlated with right ventricular diastolic filling time (*P*<0.001; *r* −0.39). Pulmonary valve presystolic ejection wave was associated with tricuspid annular plane systolic excursion (*P*<0.0001), tricuspid annular plane systolic excursion length (*P*<0.0001), right ventricular diastolic filling time (*P*<0.0001), positive end-expiratory pressure (*P*<0.0001) and peak inspiratory pressure (*P*<0.0001).

**Conclusion:**

Diastolic restrictive pattern is present in a remarkable percentage of patients with respiratory distress syndrome. Bedside echocardiography allows a mechanistic evaluation of systolic and diastolic interaction of the right ventricle.

## Introduction

Acute respiratory distress syndrome (ARDS) is associated with alveolar epithelial and microvascular endothelial injury, resulting in severe hypoxemia, decreased pulmonary compliance and increased pulmonary vascular resistance.^1,2^ The resulting increase in right ventricular (RV) afterload and different ventilatory strategies may induce RV dysfunction and, in extreme cases, acute cor pulmonale (ACP),^3^ with variable reported effects on mortality.^4^ The optimal management of ARDS remains a challenge in intensive care medicine. Lung-protective strategies, using lower end-inspiratory (plateau) airway pressure, lower tidal volumes (Vt) and higher positive end-expiratory pressure (PEEP)^5,6^ and, eventually, rescue with extracorporeal support remains the cornerstone.^6,7^

The incidence and mechanisms of RV dysfunction in the severe acute respiratory failure patient population has previously been investigated.^4^ However, data regarding incidence and outcome vary among the studies^4^ and, more importantly, the published data focused mainly on ACP, which represents the last stage of RV dysfunction.^8,9^

Aside from the literature regarding ACP, there are few studies quantifying the RV systolic function in ARDS and no data are available regarding RV diastolic function and systo-diastolic interrelation.^4^ Quantitative assessment of RV function can be performed by several methods, of which tricuspid annular plane systolic excursion (TAPSE) can be obtained routinely in critically ill patients and correlates well with RV function.^10–13^ However, there is a paucity of data regarding non-invasive indices that allow us to identify RV injury before the onset of failure.

The pulmonary valve pre-ejection wave (named PV a wave or pulmonary end-diastolic forward flow) is a sensitive index of diastolic restrictive pattern.^14^ It is detected as the forward end-diastolic pulmonary blood flow coincident with atrial systole, representing a sign of ventricular diastolic restrictive compliance, occurring when the RV end-diastolic pressure equals or exceeds pulmonary arterial diastolic pressure.^14,15^

We sought to determine the incidence of RV systolic and diastolic dysfunction, in a cohort of patients with ARDS, no features of ACP, managed with mechanical ventilation or extracorporeal support.

## Materials and methods

### Study population

We conducted a retrospective analysis cohort study of 98 consecutive patients admitted for ARDS requiring echocardiography for a period of 2 years. The study was performed at adult intensive care, Royal Brompton Hospital, London, UK. The study was approved by the local ethics committee at the Royal Brompton and Harefield NHS Foundation Trust. All selected patients were older than18 years and had a diagnosis of ARDS as defined by the Berlin definition.^16^ Patients with no echocardiographic windows, or who did not undergo echocardiography were excluded (*n*=14). Patients^12^ with ACP, defined as RV dilation with interventricular paradoxical motion leading to decreased left ventricular (LV) diastolic compliance and stroke volume reduction with severe haemodynamic impairment,^17^ were excluded as the aim of our study was to analyse features of RV dysfunction before end-stage RV failure occurs.

Patients were managed at the discretion of the treating physician with conventional protective ventilation (CV 38/98, 38%), interventional lung assistance (iLA 23/98, 23%) and veno-venous extracorporeal membrane oxygenation (VV-ECMO 37/98, 37%). The demographic data, clinical profiles, laboratory investigations and therapeutic regimens of the patients were extracted from the intensive care unit (ICU) patient data managing system (ICIP Philips Medical Systems). Patient characteristics are summarised in Table 1. The clinical data shown were recorded at the time of the echocardiography.

**Table 1.** Patients’ clinical features.

Variables	Whole	CV	iLA	VV-ECMO	*P* value
Age (years)	48 (33.7–63)	63 (44.5–72.5)	46 (29–60.5)	39 (26–51)	<0.001
APACHE II	14 (10–19)	12 (9.5–16)	11 (9–12)	20 (14.2–23)	<0.001
TV/kg (ml/kg)	3.98 (3.1–6)	6.35 (4.4–8.2)	4.6 (3.2–6.1)	3.1 (2.5–3.9)	<0.001
PIP (cmH_2_O)	28 (25–29)	27.5 (22–28)	28 (25–29)	28 (26–29)	0.233
PEEP (cmH_2_O)	11 (9–12)	12 (9–15)	11 (9–12)	11 (10–12)	0.176
pH	7.39 (7.33–7.43)	7.4 (7.36–7.45)	7.38 (7.33–7.41)	7.38 (7.32–7.42)	0.231
PaO_2_/FiO_2_	122 (93.8–174.4)	140 (112–164)	87.4 (78–104.5)	173.9 (112.3–250.2)	<0.001
PaCO_2_ (mmHg)	45.46 (41.7–52.5)	49.3 (42.6–54.6)	48.8 (44.6–52.9)	42.7 (38.2–45.8)	0.001
PV ACC T (ms)	86. 5 (75.5–98.5)	92 (80–106.5)	80 (68.2–91.5)	84 (75–106)	0.010
TAPSE (mm)	1.8 (10.15–2.1)	2 (1.7–2.3)	1.8 (1.6–1.9)	1.6 (1.35–1.9)	0.002
RVFT msec	10.9 (10.1–13.8)	11.3 (10.6–14.2)	10.9 (9.6–12.3)	–	0.409
RVET msec	8.9 (7.5–10.7)	9.1 (7.35–10.36)	9.46 (8.12–10.8)	8.45 (7.1–10.6)	0.418

The table shows the differences between the three groups of patients for the variables considered.

The values are shown as median (25th–75th percentile).

Whole: the whole population; CV: conventional ventilation; iLA: interventional lung assist; VV-ECMO: veno-venous extracorporeal membrane oxygenation; TV/kg: tidal volume per kilogram; PIP: peak inspiratory pressure; PEEP: positive end-expiratory pressure; PaCO_2_: arterial partial pressure of CO_2_; PV ACC T: pulmonary valve acceleration time; TAPSE: tricuspid annular plane systolic excursion; RVFT: right ventricular filling time adjusted for heart rate; RVET: right ventricular ejection time adjusted for heart rate.

### Echocardiography

Patients underwent transthoracic or transesophageal echocardiography as clinically indicated. All echocardiographic studies were performed within 48 hours of intensive care admission. Studies were clinically indicated by the treating physician and performed by two board-certified ICU physicians using transthoracic or transesophageal echocardiography (Philips iE33 probe S5-1 sector array transducer or Philips X7-2t, Bothell, WA 98041 USA). Echocardiographic data were retrospectively analysed offline, blinded to patient demographic and clinical characteristics. All measurements were acquired at end-expiration, and averaged on at least 3 beats when in sinus rhythm and 5–10 beats when in supraventricular arrhythmias; all the recordings were done at a paper speed of 100 mm/s with superimposed ECG trace (lead II). RV systolic and diastolic parameters were evaluated according to American Society of Echocardiography (ASE) guidelines;^18^ ventricular dimensions and volume and flow velocities were obtained using pulsed and continuous wave Doppler techniques according to the ASE and European Association of Echocardiography guidelines.^20^

Echocardiographic data analysis was performed offline by a EACVI certified operator (GT).

### RV assessment

**Figure 1.**
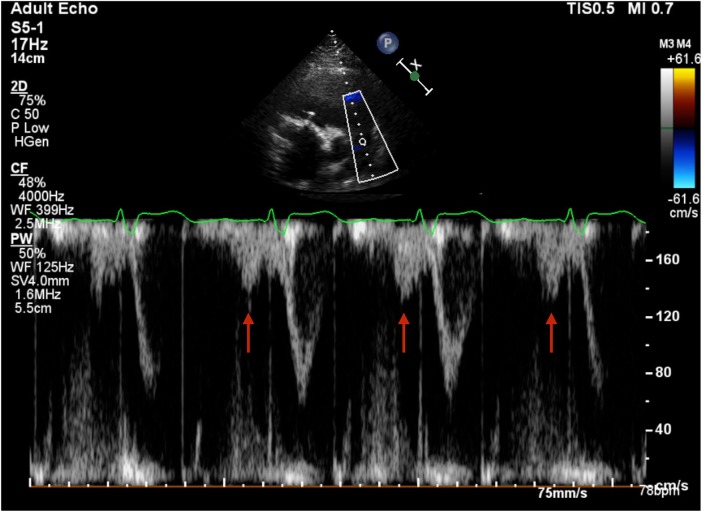
Pulse wave Doppler at the right ventricular (RV) outflow tract in short axis view sampling the flow through the pulmonary valve. Red arrows indicate the PV A wave, simultaneous at the atrial systole, preceding the RV systolic forward flow.

TAPSE was measured, in the apical four-chamber view with an M-mode cursor placed through the lateral tricuspid annulus, as the peak excursion of the tricuspid annulus (millimeters) from the end of diastole to end systole. As per guidelines, a TAPSE less than 1.6 cm was considered pathological.^20^ Moreover, TAPSE length with respect to the ECG, was also assessed and regarded as post-ejectional shortening when annular displacement peaked after the T wave on the ECG, meaning a pathological systolic contraction elongating during the proto-diastolic period.^20,21^ The pulmonary artery systolic diameter was measured in modified parasternal long axis and parasternal short axis views. Pulmonary artery flow was measured by placing the pulsed-wave Doppler sample volume at the centre of the transpulmonary valve flow. Presystolic A wave (PV a wave or pulmonary end-diastolic forward flow) is seen, when present, as an anterograde flow through the pulmonary valve in correspondence to the atrial systole (P wave at the superimposed ECG) preceding the ventricular contraction (Figure 1).

Right ventricular filling time (RVFT) was measured as the interval between the onset of the E wave to the end of the A wave using pulse wave Doppler with the cursor placed at the RV inflow in apical four-chamber view. As filling times depend on heart rate, RVFT was adjusted accordingly. RV ejection time was quantified as the interval between the onset of forward pulmonary flow and the onset of the pulmonary valve closure artefact. Furthermore, pulmonary valve acceleration time (PV ACC time), calculated as the interval between the onset of ejection and the peak flow velocity, was measured as it is a Doppler echocardiographic parameter strongly correlated with right heart catheterisation- based measures of pulmonary vascular resistance.^22,23^ Pulmonary arterial systolic pressure was determined from the tricuspid regurgitation jet velocity using the simplified Bernoulli equation adding the right atrial pressure measured directly from the central venous pressure at end expiration.^18^

### Statistical analysis

Statistical analyses were performed using SPSS (version 25; IBM Corp., Armonk, NY, USA). The normality of the data was assessed using the Shapiro–Wilk test along with inspection of histograms and QQ plots. The Kruskal–Wallis test was used for assessing differences between the three groups of patients while the Mann–Whitney test assessed differences between groups dichotomised by the presence of PV A wave. Associations between continuous variables were assessed using Spearman correlations. Intra-observer and inter-observer reliabilities were assessed by the intraclass correlation coefficient. For all tests, *P* values less than 0.05 were considered significant.

## Results

The patient characteristics are summarised in Table 1 and the aetiopathologies of ARDS are shown in Table 2.

**Table 2.** Patients’ ARDS aetiopathology.

	Whole, *n* (%)	MV, *n* (%)	iLA, *n* (%)	VV-ECMO, *n* (%)
Bacterial pneumonia	66 (67.3%)	32 (84.2%)	17 (73.9%)	17 (45.9%)
H1N1	20 (20.4%)	1 (2.6%)	6 (26.1%)	13 (35.1%)
Secondary	6 (6.1%)	4 (10.5%)		2 (5.4%)
Aspiration	3 (3%)			3 (8.1%)
Intoxication	3 (3%)	11 (2.6%)		2 (5.4%)

The percentage in the Whole column is related to the whole population. The percentage in the MV, iLA and VV ECMO columns referred to the population of the group itself.

ARDS: acute respiratory distress syndrome; *n*: number of patients; MV: conventional mechanical ventilation; iLA: interventional lung assistance; VV-ECMO: veno-venous extracorporeal membrane oxygenation.

### Reproducibility

For the parameters measured (TAPSE, TAPSE length, filling time, PV ACC time), the operator intra-observer variability was 0.996 (95% confidence interval 0.998–0.999) (see Table 3).

**Table 3.** Correlation between echocardiographic and respiratory parameters.

Echo variables	Overall (*P* value; *r*)	CV (*P* value; *r*)	iLA (*P* value; *r*)	VV-ECMO (*P* value; *r*)
**TAPSE vs. RVFT**	<0.001; 0.5	0.003; 0.47	0.053; 0.56	
**TAPSE vs. TAPSE length**	<0.001; −0.42	0.018; −0.4	0.2	0.003; −0.45
**TAPSE length vs. RVFT**	<0.001; −0.39	<0.01; −0.41	0.4	
**TAPSE vs. PEEP**	<0.001; −0.45	0.003; −0.51	0.1; −0.31	0.07; −0.29
**TAPSE vs. PIP**	<0.001; −0.34	0.02; −0.39	0.2	0.036; −0.35
**TAPSE length vs. PIP**	0.001; 0.6	< 0.001; 0.6	0.8	0.5
**RVFT vs. PIP**	<0.001; −0.6	<0.001; −0.6	0.003; −0.8	
Echo variables	Overall	CV	iLA	VV-ECMO
**TAPSE vs. RVFT**	<0.001; 0.5	0.003; 0.47	0.053; 0.56	
**TAPSE vs. TAPSE length**	0.002; −0.31	0.038;−0.34	< 0.001; −0.76	<0.001; −0.67
**TAPSE vs. PEEP**	<0.001; −0.45	0.003; −0.51	0.1; −0.31	0.07; −0.29

CV: conventional ventilation; iLA: interventional lung assistance; VV-ECMO: veno-venous extracorporeal membrane oxygenation; TAPSE: tricuspid annular plane systolic excursion; RVFT: right ventricular filling time; TAPSE length: TAPSE duration was measured in M-mode modality from the onset to the peak of systolic contraction; PEEP: positive end-expiratory pressure; PV a wave: pulmonary valve presystolic A wave; PIP: peak inspiratory pressure.

### RV systolic function

Systolic dysfunction was present in 33.6% of the patients overall in the population: nine patients (23%) in CV; two patients in iLA and 23 patients (86%) in VV-ECMO. A negative correlation was found between the reduction of TAPSE excursion and TAPSE length (*P*<0.001; *r*=−0.42), meaning that the reduction in longitudinal excursion entailed a prolongation of systolic contraction (post-systolic shortening). Furthermore, an inverse correlation resulted between TAPSE and an increase in intrathoracic pressure (PEEP and peak inspiratory pressure (PIP) *P*<0.001). A positive correlation between RVFT and TAPSE was found (*P*<0.001) whereas RVFT was inversely related to TAPSE length (*P*<0.001), meaning that the reduction in the longitudinal excursion entailed a prolongation of the contraction and a reduction in diastolic filling time.

### Pre-systolic a wave

**Figure 2.**
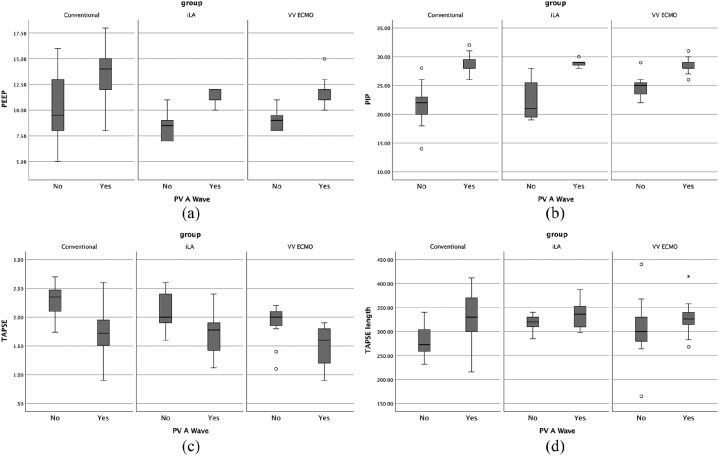
Boxplots showing the correlation among groups of PV a wave with respiratory mechanics parameter (a) positive end-expiratory pressure (PEEP); (b) peak inspiratory pressure (PIP); and systolic parameters (c) tricuspid annular plane systolic excursion (TAPSE); (d) TAPSE duration.

Presystolic a wave was present in 63 (64%) patients (16 in CV, 16 in iLA, 31 in VV-ECMO; χ^2^=13.3, *P*=0.001). PV a wave was associated with signs of impaired RV performance (TAPSE and shortened filling time) and increased afterload (higher intrathoracic pressure, PV ACC time) Figure 2. PV a wave was significantly associated with TAPSE (*P*<0.0001), TAPSE length (*P*<0.0001), RVFT (*P*<0.0001), PV ACC time (*P*<0.0001), PEEP (*P*<0.0001), PIP (*P*<0.0001) and Vt/kg (*P*<0.02) Figure 2 and Supplementary Figure 1. All the associations were maintained in CV group: TAPSE (*P*<0.001), TAPSE length (*P=*0.03), RVFT (*P*<0.001), PV ACC time (*P*<0.001), PEEP and PIP (*P*<0.001). PV a wave was shown to be related to RVFT (*P=*0.03), PEEP (*P*<0.001) and PIP (*P*<0.001) in the iLA group.

In the VV-ECMO group correlations were found between PV a wave and TAPSE (*P*=0.001), PV ACC time (*P*<0.001), PEEP and PIP (*P*<0.001) The difference of echocardiographic and ventilation variables splitting the population between patients with (presence) or without (absence) PV a wave are shown in Supplementary Table 1.

## Discussion

This is the first study exploring the RV systolic and diastolic interrelation in patients with ARDS. Echocardiography allows a comprehensive assessment of the RV function through the evaluation of systolic function (TAPSE), pulmonary vascular resistances (PV ACC time) and diastolic performance (RVFT and PV a wave). Our results show …that:

1) RV systolic dysfunciton is present in one third of patients;

2) diastolic restrictive pattern is recognisibel in more than 60% of patients;

3) it shows the close interaction between the systolic and diastolic function.

ACP has been largely investigated, and ….. The introduction of more protective ventilation and the other strategies have been demonstrated to have a beneficial impact on haemodynamics,^24^ although the occurrence of ACP is not negligible in ARDS patients ventilated with airway pressure limitation.^25^ Our patients were ventilated according to the protective ventilation strategy in term of Vt/kg, as shown in Table 1. Moreover, VV-ECMO patients appeared to be more severely ill in terms of APACHE II, RV systolic function and increased estimanted pulmonary vascular resistance (PV ACC time) (Table 1).

Our results confirm the relation previously determined on the influence of increased intrathoracic pressure on RV haemodynamics.^25–27^ Multiple studies have previously focused on the incidence, predictors and outcome of RV function in ARDS, identifying respiratory acidosis, plateau pressure, driving pressure and other respiratory variables as predictors and causal factors leading to ACP.^25–27^ However, ACP may be a relatively late phenomenon in RV dysfunction.^9^

TAPSE has been the most common parameter to define RV systolic dysfunction^10^ because it evaluates the excursion of the longitudinal fibres, revealing the contribution of the RV itself to systolic contraction.^28,29^ However, there are no data regarding post-ejectional shortening, a phenomenon related to the perfusion mismatch of the longitudinal fibres that may lead to both a reduction of longitudinal shortening and an increase in the duration of longitudinal myocardial contraction (annular displacement peaking after the T wave on the ECG).^20^ This phenomenon results in a decline of the effective proto-diastolic filling period, leading to a reduction in stroke volume and demonstrating a severe systo-diastolic interaction impairment.^30–32^ This mechanism is largely demonstrated in patients with LV dysfunction while it has never been described in the RV. We hereby report the existence of post-ejectional shortening on RV and its correlation with both reductions of TAPSE excursion and with the impairment of diastolic filling time (RVFT).

The PV A wave represents an anterograde diastolic pulmonary arterial flow, coincident with premature opening of the pulmonary valve during right atrial systole, reflecting reduced RV diastolic compliance and RV restrictive pattern at end diastole.^14,33^ Simultaneous catheter pressure monitoring has previously demonstrated that this flow occurs when the RV end-diastolic pressure equals or exceeds the pulmonary arterial diastolic pressure.^14,15^ In our population, the PV a wave was present in a consistent percentage of patients and was significantly associated with ventilation established factors that increase intrathoracic pressures and pulmonary vascular resistance (Supplementary Table 1). Interestingly, the patients in the iLA groups had minor haemodynamic impairment. One possible explanation is that those who did not exhibit hypoxic issues had less severe parenchymal impairment, with a less severe V/Q mismatch and lower pulmonary vascular resistance. Previous studies on RV dysfunction and pulmonary hypertension pathophysiology have shown that for the right ventricle to fail needs the coexistence of two conditions: an increase in afterload and a reduction of coronary perfusion.^35–38^ The increased afterload leads to significant change in RV pulmonary valve loop inducing a significant alteration in diastolic compliance.^29^ An impairment of systolic performance, besides the reduction in the longitudinal excursion, could imply a prolongation of systolic contraction reducing the effective proto-diastolic filling time, further contributing to the diastolic function impairment.^31^ The pathophysiological mechanism is demonstrated by the correlation found between the alteration of TAPSE and TAPSE duration (systolic impairment), the reduction of RVFT (effective diastolic filling) and the presence of PV a wave (diastolic compliance). This study illustrates, for the first time, the systolic and diastolic interrelation studied with echocardiography, in patients with ARDS without ACP, treated with different strategies and showing reproducible indices potentially preceding the RV failure. The recognition of early signs of RV dysfunction may have important implications in the clinical setting in terms of patient management.^38^

### Limitations

The first limitation of this study is the nature of retrospective analysis of prospectively acquired data, which limited the kind of mechanical ventilation data to be analysed. Indeed, we were unable to obtain the plateau pressure and the driving pressure at the time of the analysis as the data were retrieved from the hospital electronic data system. The second limitation is the sample size: a heterogeneous population divided into three relatively small groups without any intervention. Some of the echocardiographic parameters (RVFT) of patients treated with VV-ECMO were not analysed due to the suboptimal quality of the images acquired. The third limitation is the non-interventional nature of the study.

## Conclusion

Systolic and diastolic dysfunction is relatively common in patients with ARDS without ACP. TAPSE excursion and length, the study of RVFT and PV a wave allows the evaluation of RV systolic and diastolic performance and their interrelation elucidating the mechanism of RV impairment.
